# Age-related differences in IL-1 signaling and capsule serotype affect persistence of *Streptococcus pneumoniae* colonization

**DOI:** 10.1371/journal.ppat.1007396

**Published:** 2018-10-31

**Authors:** Kirsten Kuipers, Kristen L. Lokken, Tonia Zangari, Mark A. Boyer, Sunny Shin, Jeffrey N. Weiser

**Affiliations:** 1 Department of Microbiology, New York University School of Medicine, New York, New York, United States of America; 2 Department of Microbiology, University of Pennsylvania Perelman School of Medicine, Philadelphia, Pennsylvania, United States of America; The University of Alabama at Birmingham, UNITED STATES

## Abstract

Young age is a risk factor for prolonged colonization by common pathogens residing in their upper respiratory tract (URT). Why children present with more persistent colonization is unknown and there is relatively little insight into the host-pathogen interactions that contribute to persistent colonization. To identify factors permissive for persistent colonization during infancy, we utilized an infant mouse model of *Streptococcus pneumoniae* colonization in which clearance from the mucosal surface of the URT requires many weeks to months. Loss of a single bacterial factor, the pore-forming toxin pneumolysin (Ply), and loss of a single host factor, IL-1α, led to more persistent colonization. Exogenous administration of Ply promoted IL-1 responses and clearance, and intranasal treatment with IL-1α was sufficient to reduce colonization density. Major factors known to affect the duration of natural colonization include host age and pneumococcal capsular serotype. qRT-PCR analysis of the uninfected URT mucosa showed reduced baseline expression of genes involved in IL-1 signaling in infant compared to adult mice. In line with this observation, IL-1 signaling was important in initiating clearance in adult mice but had no effect on early colonization of infant mice. In contrast to the effect of age, isogenic constructs of different capsular serotype showed differences in colonization persistence but induced similar IL-1 responses. Altogether, this work underscores the importance of toxin-induced IL-1α responses in determining the outcome of colonization, clearance versus persistence. Our findings about IL-1 signaling as a function of host age may provide an explanation for the increased susceptibility and more prolonged colonization during early childhood.

## Author summary

During early childhood, opportunistic pathogens are often carried in the upper respiratory tract (URT) for prolonged periods of time. Why young children experience more persistent carriage is unclear and there is little understanding of host-bacteria interactions that affect persistence, especially in infants. Here, we utilized an infant mouse model of *Streptococcus pneumoniae* colonization, a common pathogen of the infant URT, that persists for several months. We identified that clearance is dictated by bacterial expression of a single pneumococcal toxin, pneumolysin, and by the host response via a single cytokine, IL-1α, that activates IL-1 signaling. Absence of either of these factors led to increased persistence of *S*. *pneumoniae*. We discovered that the infant URT shows repression of IL-1 signaling compared to adults. Our study presents new insight into the importance of IL-1 signaling in clearance of persistent URT carriage and may provide an explanation why infants present with more persistent carriage by common URT pathogens.

## Introduction

Respiratory tract infections remain a leading cause of childhood morbidity and mortality worldwide. In 2016, pneumonia accounted for 16% of all deaths in children younger than five years of age [1]. Epidemiological studies have demonstrated young age to be a risk factor for colonization of common respiratory tract pathogens, including *Streptococcus pneumoniae* (pneumococcus) [2, 3]. Carriage of respiratory pathogens on the mucosal surface of the upper respiratory tract (URT) is a necessary first step in the pathogenesis of all invasive pneumococcal diseases [4]. Clinically, 25–65% of healthy children are colonized with *S*. *pneumoniae* in the URT, in contrast to <10% of adults [5, 6]. Furthermore, pneumococcal colonization of the URT is prolonged in young children compared to adults [7–9]. This increased persistence of *S*. *pneumoniae* in young children is suggestive of a more commensal relationship between bacteria and host. The decline in pneumococcal carriage beyond childhood correlates with a general decrease in the complexity and density of the URT flora with increasing age [10]. Why young children present with more prolonged colonization by bacteria residing on the mucosal surfaces of the URT, like *S*. *pneumoniae*, is unknown.

Most pneumococci are encapsulated with one of >95 antigenically distinct capsular polysaccharides (CPS), the determinant of serotype. In animal models of URT colonization, expression of CPS, which inhibits phagocytic clearance, is necessary for colonization duration to last more than a few days [11]. A recent bacterial GWAS study from a large infant-mother cohort found capsular serotype to be the major determinant of carriage duration [12].

Other studies in adult mouse models of pneumococcal colonization have shown that expression of its other major virulence factor, the toxin pneumolysin (Ply), is inversely correlated with carriage duration [13, 14]. Ply is a cytolysin that inserts into cholesterol-containing membranes where it oligomerizes to form large pores, however it is an unusual toxin as it lacks an N-terminal secretion signal sequence or other secretion mechanism [15, 16]. The release of Ply, therefore, requires bacterial lysis. For example, following uptake and degradation by professional phagocytes, Ply is released into the phagosome where, by forming pores into the membrane of the phagosome, it allows microbial products to access the host cytosol. Cytosolic access activates inflammatory pathways that signal the recruitment and activation of additional phagocytes that eventually promote mucosal clearance [14, 17–19]. These effects of Ply also eventually result in cell lysis and death.

Although many studies have focused on the molecular mechanisms that drive initial host responses to acute infection, there is little knowledge of host and bacterial factors permissive for persistent colonization, particularly during infancy. This study was undertaken to elucidate host and bacterial molecular mechanisms facilitating persistent colonization using *S*. *pneumoniae* as a model pathogen. Using the variables of host age, capsule serotype and pneumolysin expression, we assessed how pneumococcal colonization is either cleared or persists. We demonstrate that Ply-mediated mucosal IL-1 signaling via the release of IL-1α is critical for clearance of otherwise persistent colonization and that infants are deficient in IL-1 signaling compared to adults. Our observations of reduced IL-1 signaling early in life provides mechanistic insight into the altered dynamics of pneumococcal colonization with age.

## Materials and methods

### Ethics statement

All animal studies were performed in compliance with the federal regulations set forth in the Animal Welfare Act (AWA), the recommendations in the Guide for the Care and Use of Laboratory Animals of the National Institutes of Health, and the guidelines of the New York University School of Medicine and the University of Pennsylvania Institutional Animal Use and Care Committee. All protocols used in this study were approved by the Institutional Animal Care and Use Committee at the New York University School of Medicine (protocol #160622 and #161219) and the University of Pennsylvania (protocol #804928).

### Bacterial stains and culture

*S*. *pneumoniae* isolates serotypes 4 and 23F and previously described isogenic Ply mutants were used throughout the study [18, 20, 21]. In the serotype 4 background, these mutants included an unmarked, inframe deletion of *ply*, a point mutant expressing Ply_W433F_, which is deficient at oligomerization to form pores after membrane insertion, and a corrected mutant where the intact gene was restored (*ply*+). In the serotype 23F background, we used a mutant expressing Ply_TL→AA_, which fails to bind to cholesterol and insert into membranes [17, 18]. The genotype of mutants was confirmed by sequence analysis of PCR products. The phenotype of all strains was confirmed using a previously described horse erythrocyte lysis assay [17, 18]. The hemolytic activity of Ply-deficient and point mutants was less than 1% of WT levels. The serotype 4 and 23F parent strains showed no differences from one another in hemolytic activity or Ply protein levels as measured in Western blots. Type 4 and 23F isogenic capsule-switch strains 23F^4^ (23F genetic background expressing the serotype 4 capsule) and 4^23F^ (4 genetic background expressing the serotype 23F capsule) were used to study serotype-dependent effects and were previously described [22]. All pneumococcal strains were grown in tryptic soy (TS) broth (BD) at 37°C to an optical density of 1.0 at 620 nm (OD_620_). Alternatively, pneumococci were incubated on TS agar plates supplemented with 100 μl of catalase (30,000 U/ml; Worthington Biomedical) and streptomycin (200μg/ml) at 37° in 5% CO_2_ overnight.

### Mouse studies

Wild-type C57BL/6J mice and congenic knockout mice (*muMT*^*-/-*^, *Tlr2*^*-/-*^, *Nod2*^*-/-*^, *Ifnar*^*-/-*^, *Ccr2*^*-/-*^, *Il1r*^*-/-*^, *Nlrp3*^*-/-*^) were obtained from Jackson Laboratories (Bar Harbor, ME), and bred and maintained in a conventional animal facility. The *Il1a*^*-/-*^ and *Il1b*^*-/-*^ mice were generously provided by Dr. Yoichiro Iwakura at the Tokyo University of Science [23]. Pups were housed with a dam until weaned at the age of 3.5 weeks. During colonization, all mice appeared healthy and demonstrated normal weight gain similar to uninfected controls.

Pups at day 4 of life were infected with 10^3^−10^4^ CFU of *S*. *pneumoniae* in 3 μl PBS by intranasal (i.n.) instillation divided over both nares without the use of anesthesia. At the time points indicated following challenge, mice were euthanized by CO_2_ asphyxiation followed by cardiac puncture. The trachea was lavaged with 200 μl sterile PBS collected from the nares to determine URT colonization density of *S*. *pneumoniae*. A second URT lavage with 600 μL RLT lysis buffer (Qiagen) + 1% β-mercaptoethanol was performed to obtain host RNA from the URT epithelia for gene expression analyses.

### RNA-sequencing

Uninfected infants, serotype 23F wild-type and *ply-* mutant infected pups (aged 1 week) were euthanized 3 days post-challenge and a URT lavage with RLT lysis buffer was obtained. RNA was extracted according to manufacturer’s directions (RNeasy kit, Qiagen) and five replicates per age group were subjected to RNA sequencing (RNA-seq) using Hi-seq and the raw fastq reads were aligned to mm10 mouse reference genome using STAR aligner [24]. Fastq Screen was used to check for any contaminations in the samples and Picard RNA-seqMetrics was used to obtain the metrics of all aligned RNA-Seq reads. *featureCounts* [25] was used to quantify the gene expression levels. All alignments and read metrics are summarized in the supplementary data. The raw gene counts data were used for further differential expression analysis. To identify the differentially expressed genes (DEGs), *DESeq2* R package [26] was used and results were subsequently analyzed using the online annotation tool DAVID Bioinformatics Resources [27]. The resulting genes with FDR < 0.05 were considered significant. Heatmaps were generated using *pheatmap* R package. RNAseq data are made available in the GEO repository under project accession number GSE116604.

### ELISA

Anti-pneumococcal IgG titers were assessed in serum from uninfected and infected mice using whole bacterial cells as the capture antigen, as described previously with some adjustments [28]. Type 23F strain was grown to an OD_620_ of 1 and washed with PBS. Pneumococcal cells were diluted to final OD_620_ of 0.1 in coating buffer (0.015 M Na_2_CO_3_, 0.035 M NaHCO_3_). Microtiter plates (2HB, Immunolon) were coated with 100 μL suspension/well at 4°C overnight. The next day, the plates were blocked with 1% bovine serum albumin (BSA) in PBS at room temperature for 1 hour, after which the plates were incubated with serial serum dilutions in PBS at 4°C overnight. Peroxidase-conjugated goat anti-mouse IgG (Jackson Immuno Research Laboratories) was applied and plates were incubated at 37°C for 1.5h. Between incubation steps, plates were washed three times with 0.05% Brij-35 (Thermo Fisher Scientific) in PBS. Plates were developed using 100 μL/well substrate o-phenylenediamine dihydroxychloride (OPD, Thermo Fisher Scientific, 1 tablet in 7.5 ml H_2_O with 7.5 μL 30% H_2_O_2_) and incubated at room temperature for 30 minutes in the dark. Reactions were stopped with 50 μl/well of 2M H_2_SO_4_ and the absorbance was measured at 492 nm. Serum IgG titers were determined by calculating the dilution at which the absorbance was equal to an OD_492_ of 0.1.

### Quantitative RT-PCR

RNA extraction (RNeasy, Qiagen) from nasal RLT lavages and subsequent cDNA generation using High Capacity cDNA Reverse Transcriptase Kit (Applied Biosystems, Thermo Fisher Scientific) were performed as per manufacturer’s instructions. Reaction samples contained ~10ng cDNA and 0.5 μM primers in Power SYBR^®^ Green PCR Master Mix (Applied Biosystems, Thermo Fisher Scientific) and samples were tested in duplicate. qRT-PCR reactions were run in a 384 well plate (Bio-Rad) using CFX384^™^ Real-Time System (Bio-Rad). Expression of *Gapdh* was an internal control and fold change in gene expression was quantified according to the ΔΔCt method [29]. The primer sequences used in this study are indicated in S1 Table.

### Recombinant pneumolysin purification and treatment

As previously described, recombinant pneumolysin (PLY) and pneumolysoid (PdB), bearing the Ply_W433F_ point mutation, were expressed in *E*. *coli*, after which bacterial cells were lysed using sonication [17, 30]. The His-tagged proteins from cell suspensions were purified on a HisTrap column (GE Healthcare) by FPLC using Äkta Start (GE Healthcare) using binding buffer (20 mM NaH_2_PO_4_, 500 mM NaCl, 20 mM Imidazole, pH 7.4) and elution buffer (20 mM NaH_2_PO_4_, 500 mM NaCl, 500 mM Imidazole, pH 7.4). Cell lysates were loaded on the column after which washing was performed to eliminate binding by non-specific proteins. His-tagged protein was recovered from the column by running a 70–100% gradient of elution buffer over the column after which the protein fractions were collected. Protein desalting was done using Amicon^®^ Ultra-15 30 Kb centrifuge filters (Millipore). Protein concentration was assessed by Bradford protein assay (Bio-rad) and hemolytic activity was confirmed by horse erythrocyte lysis assay.

Pups infected with the serotype 4 *ply*- mutant at age 4 days received a first dose of PLY or PdB 1 day after challenge. Infants were treated with 100 ng protein/dose in PBS given by i.n. administration and control pups received PBS alone. Pups received daily treatment for 4 consecutive days and 5 days post-challenge pups were euthanized to determine pneumococcal colonization density and gene expression.

### Treatment with IL-1 recombinant protein

Recombinant IL-1α (Peprotech) and IL-1β (eBioscience) protein was administered daily via i.n. treatment to 4-day old pups infected with the serotype 4 *ply*- mutant strain from day 1 through 3 post-challenge. Pups received 10 or 100 ng protein in 3 μl PBS, whereas PBS alone was administered to control animals. At day 4 post-challenge, infant mice were euthanized to obtain URT lavages to assess colonization density and for gene expression analyses.

### Statistical analyses

All statistical analyses were performed using GraphPad Prism 7.0 (GraphPad Software Inc., SanDiego, CA). Colonization density values were transformed into logarithmic values. Unless otherwise specified, differences in colonization and gene expression were determined using t-test or one-way Anova with Sidak’s multiple comparison test.

## Results

### Lack of pneumolysin toxin allows persistent colonization of *S*. *pneumoniae*

**Fig 1:**
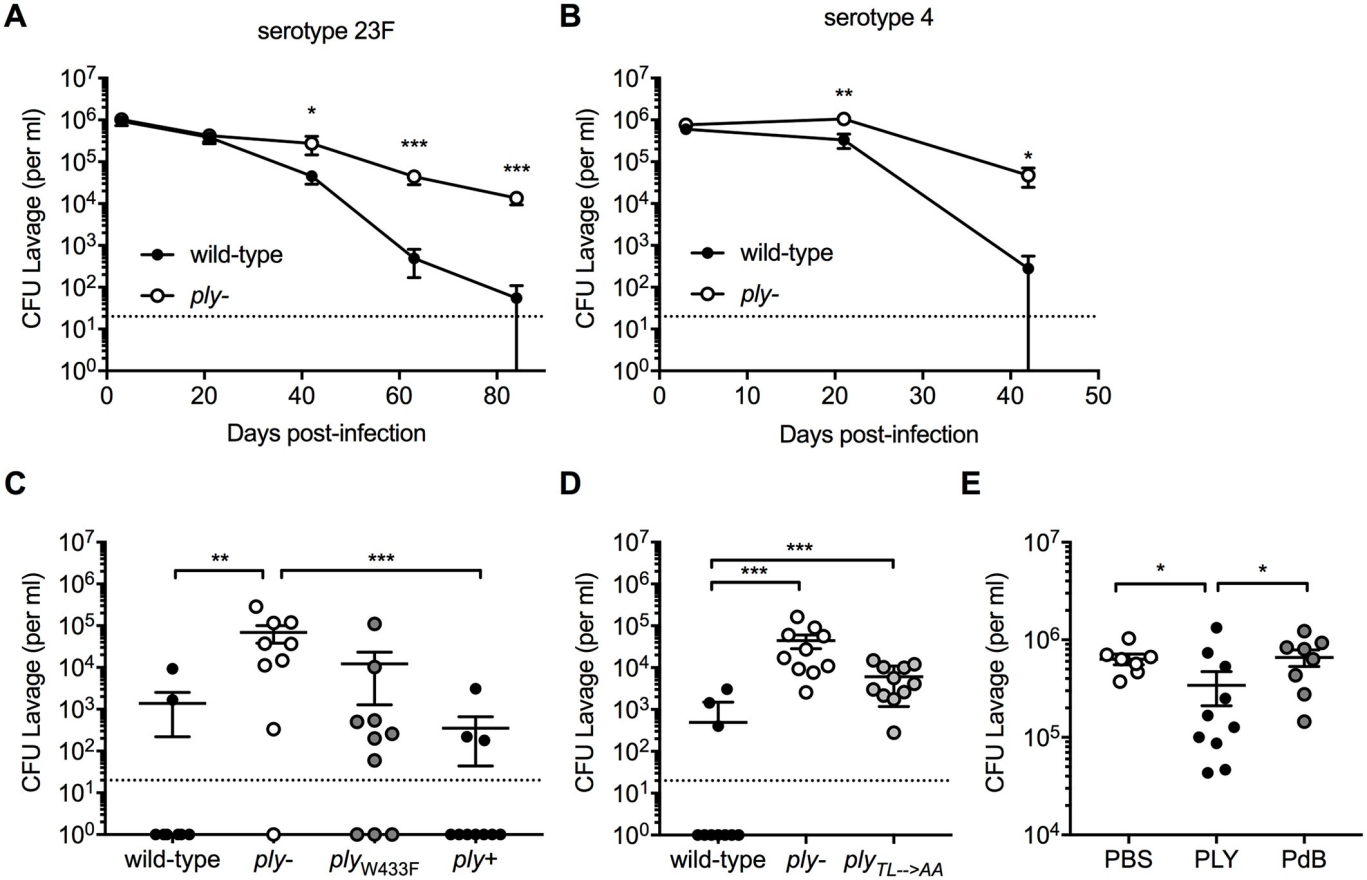
Lack of pneumolysin toxin allows for persistence of pneumococcal colonization. On day 4 of life, pups were i.n. infected with wild-type and Ply-deficient mutant (*ply-*) *S*. *pneumoniae* strains. Colonization density was assessed every 3 weeks by culture of URT lavages until clearance of the wild-type strain. Clearance of the serotype (A) 23F and (B) 4 was monitored until 9 or 6 weeks post-challenge, respectively. Pneumococcal colonization density of (C) serotype 4 strains: wild-type, *ply-*, *ply*_*W433F*_ (deficient pore formation), and corrected mutant *ply+* at 6 weeks post-infection, and of (D) serotype 23F wild-type, *ply-* and *ply*_*TL→AA*_ (deficient cholesterol binding) at 9 weeks after infection. (E) Pups infected with the serotype 4 *ply-* received daily treatments with 100 ng recombinant PLY or PdB (*ply*_*W433F*_) starting at 24 hours until 4 days after infection. Control animals received PBS dosing. Colonization density of *S*. *pneumoniae* was determined 5 days post-challenge. Groups represent n = 7–11 animals with mean ±SEM. Dotted line represents the lower limit of detection. Significance is indicated by *, P < 0.05; **, P < 0.01, ***, P < 0.001.

Infant mice have an increased density and duration of pneumococcal carriage compared to adult mice, recapitulating the effect of age during natural colonization [31]. To determine whether loss of pneumolysin (*ply-*) prolongs pneumococcal colonization in infant mice, pups were challenged i.n. at day 4 of age with a serotype 23F isolate or *ply*-deficient mutant. Clearance of the wild-type strain required approximately 9 weeks by which time the pups had become adults (Fig 1A). In contrast, at this time point colonization of the *ply-* strain remained at >10^4^ CFU/ml. To determine whether the effect of pneumolysin on colonization was independent of serotype, we infected pups i.n. at day 4 of age with a serotype 4 isolate or *ply*-deficient mutant. Although the wild-type serotype 4 isolate was cleared earlier than the wild-type 23F strain, at approximately 6 weeks post-challenge, clearance of the serotype 4 *ply*-deficient strain was significantly delayed compared to the wild-type serotype 4 strain (Fig 1B). Correction of the *ply* deletion (*ply*+) restored clearance to wild-type levels confirming the contribution of pneumolysin in attenuating colonization duration (Fig 1C). Together these results indicate that expression of pneumolysin significantly decreases duration of pneumococcal colonization. At 6 weeks post-challenge, most mice remained colonized with the non-hemolytic point mutant (W_433_F), suggesting that the pore forming activity of pneumolysin contributes to clearance of pneumococcus (Fig 1C). The requirement of toxin-host cell interaction was further supported by persistent colonization of the *ply*_*TL→AA*_ mutant, which expresses a toxin deficient in a prior step, attachment to cholesterol, in the serotype 23F background (Fig 1D). Additionally, i.n. dosing of recombinant pneumolysin (PLY), but not the toxoid containing the W_433_F modification (PdB), accelerated clearance of the *ply*-deficient mutant (Fig 1E). We concluded that pneumolysin was both necessary and sufficient for pneumococcal clearance in infant mice.

### Host responses involved in pneumolysin-mediated clearance

**Fig 2:**
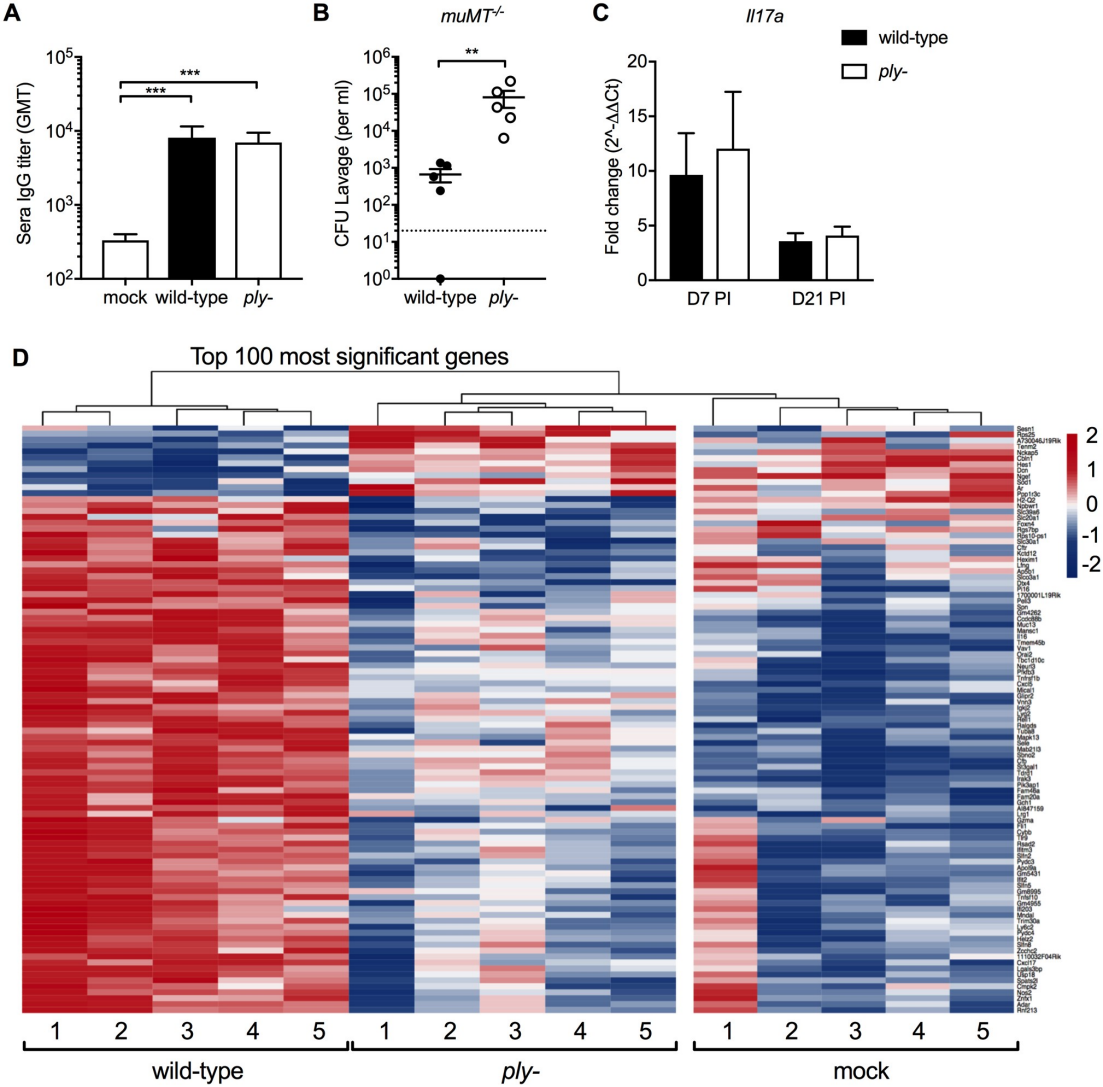
Host responses involved in pneumolysin-mediated clearance. Pups aged 4 days were infected with serotype 23F wild-type or *ply-*. (A) Sera IgG titers as determined by whole cell ELISA at 9 weeks post-challenge compared to age-controlled uncolonized (mock) mice. (B) Pneumococcal colonization density assessed in *muMT*^*-/-*^ mice at 9 weeks post-challenge. (C) URT *Il17a* expression by qRT-PCR at day 7 (D7) and 21 (D21) post-infection. Fold-change was calculated from age-corrected mock-infected controls. Groups represent n = 5–11 animals with mean ±SEM. Dotted line represents the lower limit of detection. Significance is indicated by **, P < 0.01; ***, P < 0.001. (D) At day 21 after infection, five wild-type, *ply-*, and mock challenged pups received RLT lavages to obtain RNA for RNA-seq analysis. Heat-map of top 100 differentially regulated genes comparing wild-type to *ply-* demonstrating relative gene expression in log2 foldchange with increased expression in red and decreased expression in blue.

Clearance of pneumococcal colonization is generally thought to require adaptive immune responses consisting of specific immunoglobulin or T_H_17 immunity [32–35]. Colonization increased levels of pneumococcal-specific IgG, however these did not differ between wild-type and *ply*-deficient mutant colonization (Fig 2A). Moreover, mice deficient in the generation of specific antibody (*muMT*^*-/-*^) cleared colonization of wild-type and *ply-* strain similar to C57BL6 wild-type mice (Fig 2B). *Il17a* expression was increased during colonization, but expression did not differ between WT and the *ply*-deficient mutant at day 7 and 21 post-challenge (Fig 2C). These observations suggested that the increased persistence in infants may largely depend on innate immune responses.

To more broadly explore how pneumolysin mediates clearance of otherwise persistent colonization, we performed an RNA-Seq screen on RNA isolated from URT lavages of infant mice infected with the wild-type or *ply-* deficient mutant at 21 days post-challenge, the time point at which clearance of the serotype 23F isolate is initiated. Large numbers of host genes were significantly affected by pneumolysin expression. For the 100 most differentially expressed genes (Fig 2D), pneumococcal colonization without pneumolysin resembled mock-infected controls indicating pneumolysin affects critical host responses that promote clearance.

### IL-1 signaling is essential for clearance of persistent colonization

**Fig 3:**
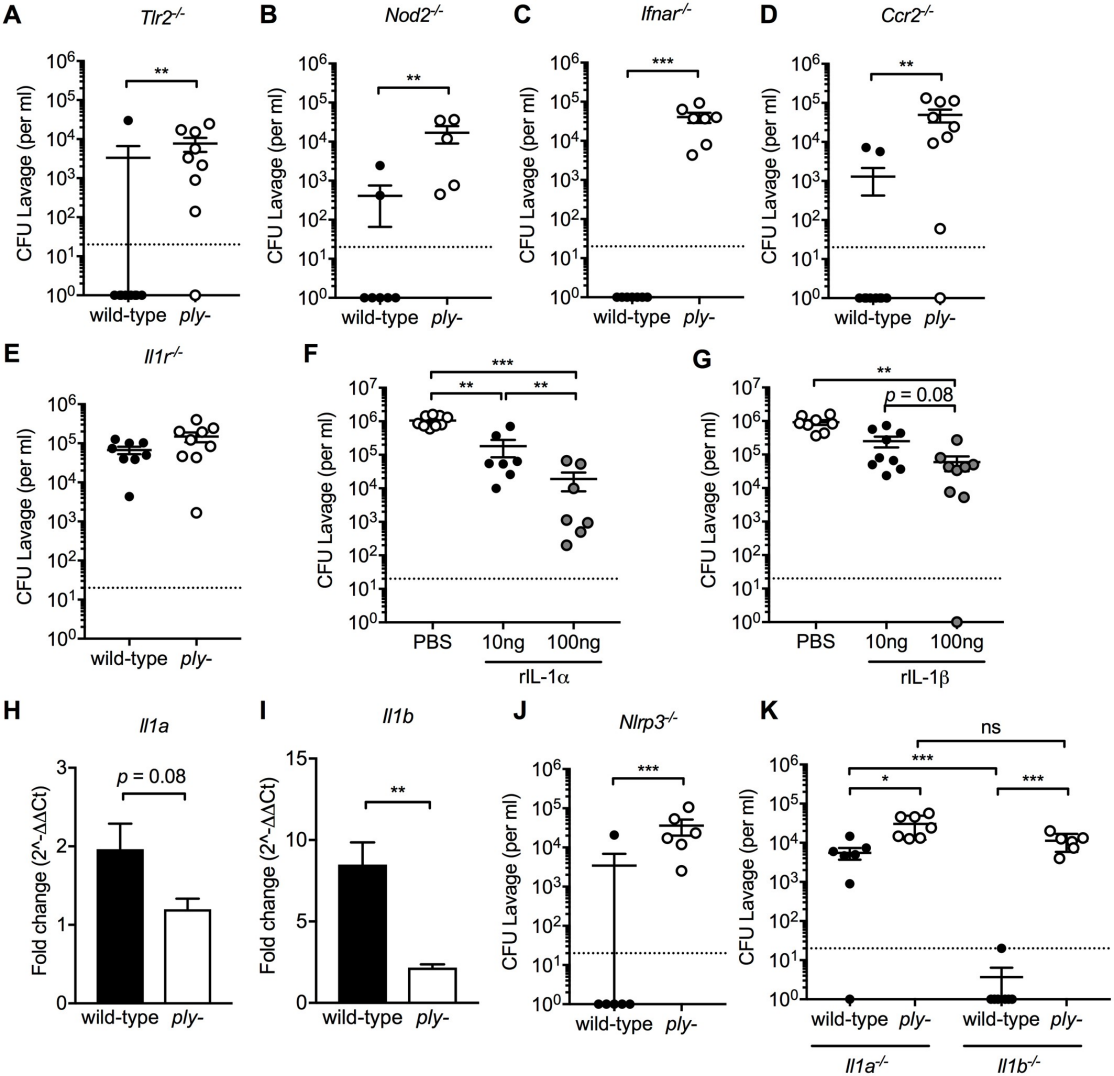
Lack of IL-1 signaling is essential for pneumococcal persistence. Pups aged 4 days were infected with serotype 23F wild-type or *ply-*. (A-D) Pneumococcal colonization density at 9 weeks post-infection in (A) *Tlr2*^*-/-*^, (B) *Nod2*^*-/-*^, (C) *Ifnar*^*-/-*^, and (D) *Ccr2*^*-/-*^, *and (E) Il1r*^*-/-*^ mice. (F-G) Starting 24 hours following challenge, pups infected with the serotype 4 *ply-* received daily i.n. treatment with 10 or 100 ng of recombinant (F) IL-1α or (G) IL-1β for 3 consecutive days. Control pups received PBS treatment and pneumococcal colonization density was determined at 4 days post-infection. (H-I) At 21 days post-challenge, the URT of infants was lavaged to assess expression of mucosal (H) *Il1a* and (I) *Il1b* by qRT-PCR and calculated as fold-change compared to mock-challenged age-controlled animals. (J-K) At 9 weeks after inoculation, pneumococcal colonization density was determined in (J) *Nlrp3*^*-/-*^, (K) *Il1a*^*-/-*^ and *Il1b*^*-/-*^ mice. Groups represent n = 5–11 animals with mean ±SEM. Dotted line represents the lower limit of detection. Significance is indicated by *, P < 0.05; **, P < 0.01, ***, P < 0.001.

We then investigated the role of innate immune signaling through TLR2, NOD2, IFNAR and IL-1R. These sensors of pneumococcal PAMPs or cytokines are expressed at significantly higher levels in wild-type versus mock infected mice in the RNA-Seq analyses and were previously shown to contribute to pneumococcal clearance in adult mouse models [13, 14, 19, 36]. Colonization of the wild-type and *ply-* mutant was assessed at 9 weeks post-challenge in wild-type versus knockout mice (Fig 3A–3C and 3E). Only for *Il1r*^*-/-*^ pups colonization of the wild-type strain persisted and was indistinguishable from the *ply*-mutant.

IL-1α and IL-1β are two cytokines released under different circumstances that exert identical biological effects downstream of the IL-1 receptor binding [37]. Previous *in vitro* studies showed that pneumolysin promotes release of both IL-1α and IL-1β by *S*. *pneumoniae* infected macrophages [19, 38]. Daily i.n. dosing of recombinant IL-1α or IL-1β accelerated clearance of *ply*- colonized pups (Fig 3F and 3G). Quantitative RT-PCR (qRT-PCR) of RNA isolated from URT lavages showed that pneumococcal colonization minimally upregulated *Il1a* expression, whereas the difference in *Il1a* expression between wild-type and *ply-* strains did not reach significance (Fig 3H). This was not unexpected, since unlike IL1β, the damage associated molecular pattern (DAMP) IL-1α is constitutively expressed in epithelial, endothelial and hematopoietic cells in the airway epithelium, although its transcription can be upregulated by strong inflammatory stimuli [39, 40] In contrast, colonization of the wild-type, but not the *ply*-mutant, significantly increased expression of *Il1b* in the URT (Fig 3I). Although IL-1α levels in URT lavages were detectable by ELISA, there was no detectable difference between infected and uninfected pups. For IL-1β, its levels in the highly diluted URT lavages were below the level of detection in all treatment groups despite using a sensitive ELISA. IL-1β is generated from pro-IL-1β as a result of caspase 1 activation by the inflammasome. *In vitro* and *in vivo* models using *S*. *pneumoniae* or pneumolysin support a role of the NLRP3 inflammasome in this process [41–44]. However, persistence of the wild-type strain was unaffected in *Nlrp3*^*-/-*^ pups (Fig 3J), suggesting a role for another inflammasome or non-canonical pathway for IL-1β processing or redundant role for IL-1β [37]. To determine the roles of individual IL-1 cytokines in clearance, we infected IL-1α and IL-1β deficient pups with the wild-type *S*. *pneumoniae* or the *ply*-deficient mutant (Fig 3K). Loss of IL-1α, but not IL-1β, resulted in impaired clearance of the wild-type strain. Colonization density at 9 weeks post-challenge of the wild-type strain was slightly less (<1 log) compared to its *ply-* mutant in *Il1a*^*-/-*^ infant mice (Fig 3J), suggesting that factors other than IL-1α play a minor role in Ply-mediated clearance. We concluded that IL-1 signaling is necessary and sufficient to prevent persistent colonization of wild-type pneumococci in infant mice, with a dominant role for IL-1α.

### Pathways involved in IL-1-mediated clearance driven by pneumolysin

**Fig 4:**
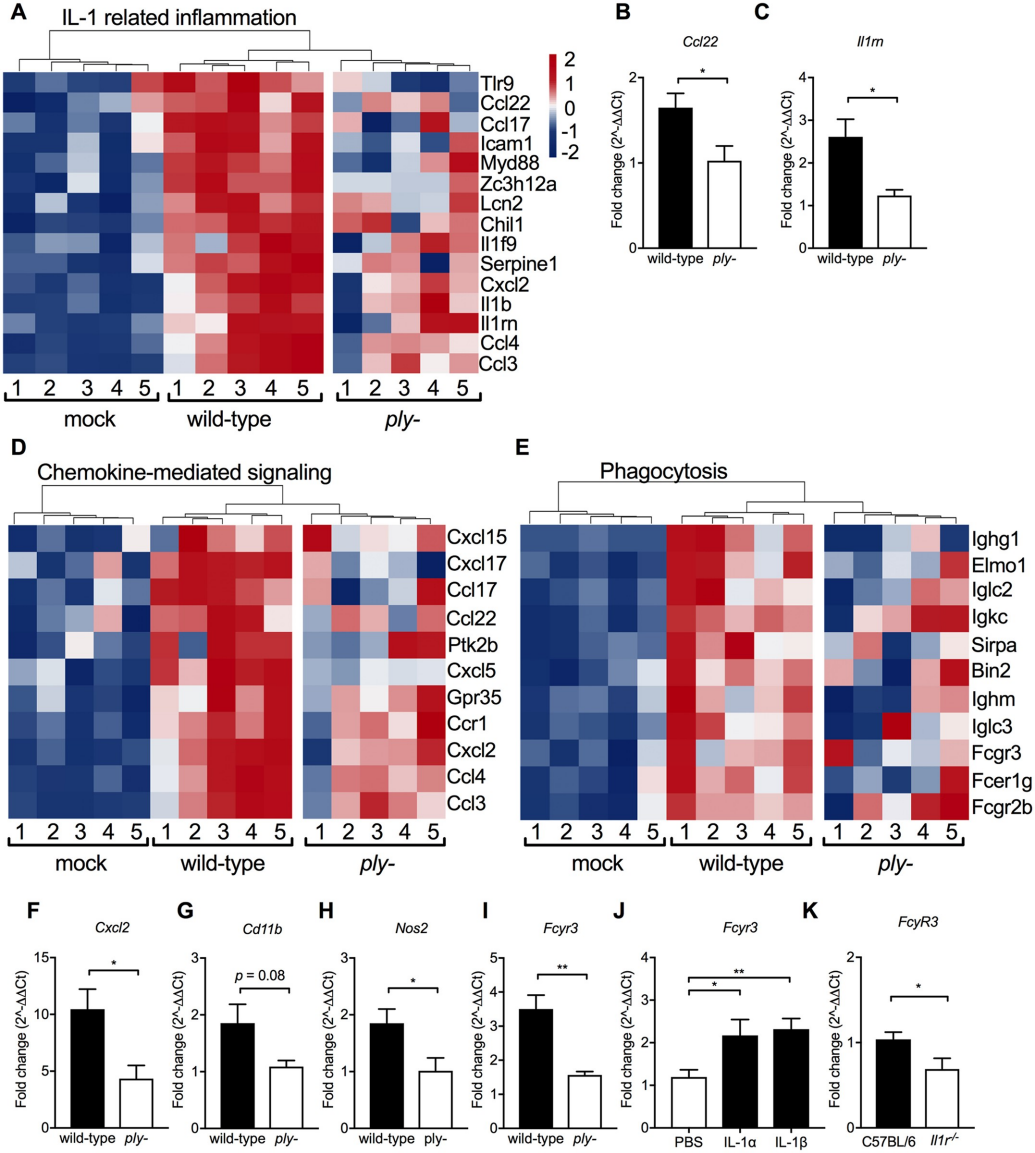
Pathways involved in clearance of pneumococcal colonization. At day 4 of life, pups were i.n. infected with serotype 23F wild-type or *ply-* and URT was lavaged with RLT to obtain RNA for RNA-seq comparisons. RNA-seq heatmaps show gene expression of the comparison wild-type colonized versus mock controls with relative gene expression in log2 fold change demonstrating increased expression in red and decreased expression in blue. Expression of these genes during *ply-* colonization is shown for comparison. (A) Heat-map illustrating gene expression in *IL-1 related inflammation* pathways. qRT-PCR was used to measure expression of (B) *Ccl22* and (C) *Il1rn* that was calculated as fold-change compared to mock-challenged age-controlled animals. (D-E) Heatmaps showing go-term clusters (D) *Chemokine-mediated signaling* and (E) *Phagocytosis* that are significantly upregulated following pneumococcal colonization. qRT-PCR was used to measure expression of (F) *Cxcl2*, (G) *Cd11b*, (H), *Nos2*, and (I) *Fcyr3* at 21 days post-inoculation. Fold-change compared to mock-challenged age-controlled animals. (J) Fold change in *Fcyr3* expression in serotype 4 *ply-* colonized pups following IL-1α and IL-1β dosing as compared to PBS-treated control pups at 4 days post-challenge. (K) Fold change in *Fcyr3* expression in 23F colonized C57BL6 and *Il1r*^*-/-*^ pups at 21 days post-inoculation. Groups represent n = 7–11 animals with mean ±SEM. Significance is indicated by *, P < 0.05; **, P < 0.01.

The RNA-Seq screen on RNA isolated from infant URT lavages at 21 days post-challenge confirmed the importance of IL-1 signaling in clearance of persistent colonization. Pathway analyses demonstrated increased expression of gene cluster *Signal Transduction Through IL-1 Receptor* following colonization with *S*. *pneumoniae*, while these gene transcripts were less stimulated in absence of pneumolysin (Fig 4A). This was confirmed by qRT-PCR showing decreased *Ccl2* and *IL1rn* expression during *ply-* compared to wild-type colonization at 21 days (D21) post-challenge (Fig 4B and 4C). Furthermore, the RNA-Seq data revealed two major pathway upregulated during colonization potentially contributing to clearance of pneumococcal colonization, *Chemokine-mediated signaling* pathway and *Phagocytosis* (Fig 4D and 4E). These pathways were also less activated during colonization by the *ply-* deficient strain. Clearance of pneumococcal colonization requires chemokine-dependent recruitment of professional phagocytes into the lumen of the URT and the expression of multiple chemokines were upregulated by colonization [19, 34]. There was, however, no non-redundant role for CCR2 in clearance in infant mice, although this chemokine receptor was previously shown to be important in clearance in adult mice (Fig 3D) [14]. By comparing transcript levels using qRT-PCR on RNA isolated from URT lavages, we confirmed that representative genes revealed in these pathways analyses involved in phagocyte recruitment (chemokine Cxcl2), activation (leukocyte integrin CD11b), activity (nitric oxide synthase, Nos2), and regulation (Fc gamma receptor, FcγR3) were all increased in expression during wild-type compared to *ply-* colonization (Fig 4F–4I). Additionally, i.n. administration of recombinant IL-1α and IL-1β were both sufficient to enhance transcription of FcγR3 (Fig 4J). As was the case for *ply-* infection, transcription of FcγR3 following wild-type colonization was also impaired in *Il1r*^*-/-*^ mice (Fig 4K). These observations were consistent with a clearance mechanism dependent on professional phagocytes that requires IL-1 signaling.

**Fig 5:**
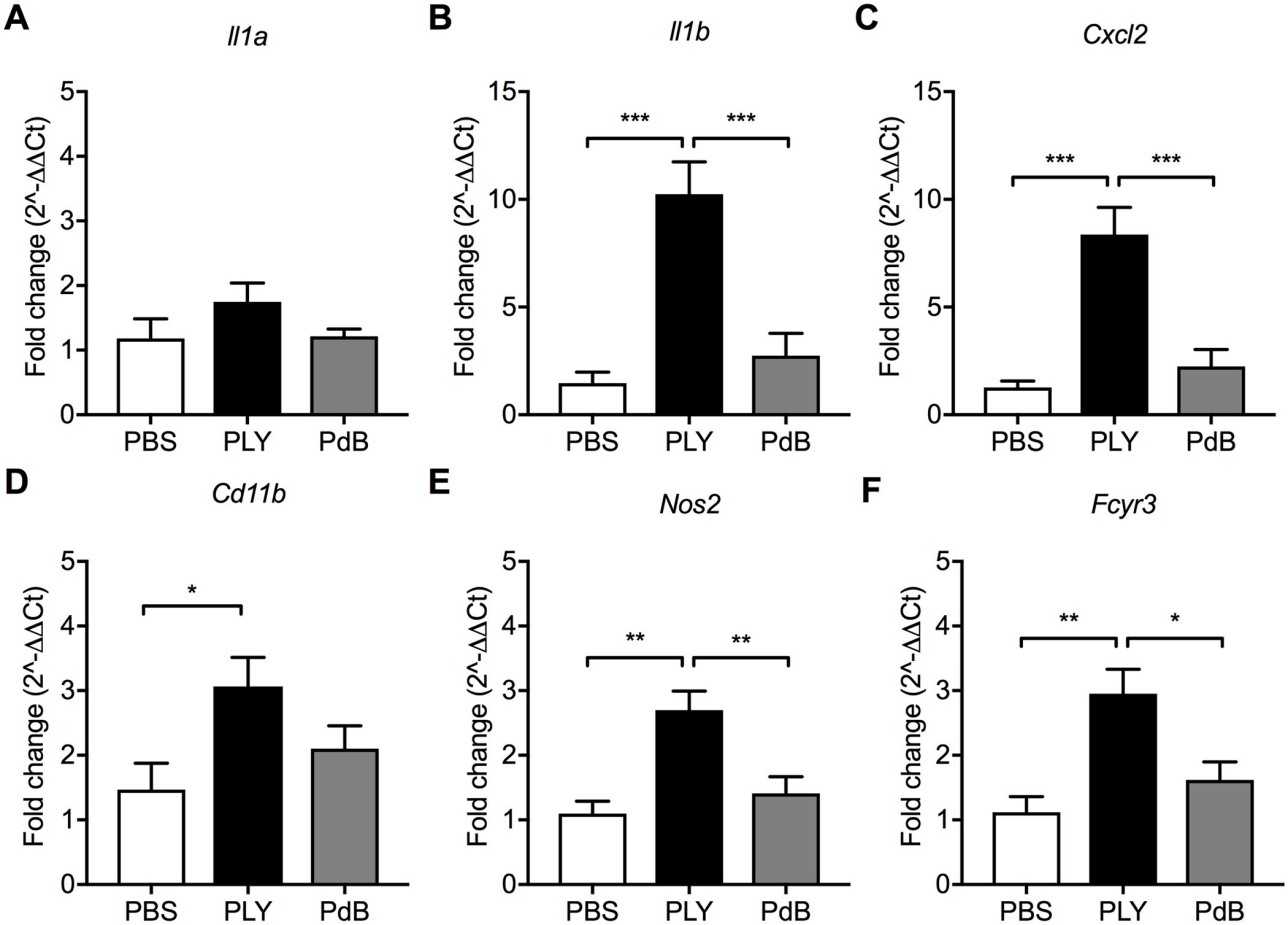
Pneumolysin pore-formation drives host responses. Pups colonized with the serotype 4 *ply-* received daily treatments with 100 ng recombinant Ply (PLY) or toxoid PdB (W_433_F) starting at 24 hours until 4 days after inoculation. Control animals received PBS dosing. The URT of the pups was lavaged to obtain RNA for qRT-PCR analysis at 5 days post-challenge. Fold change in expression of (A) *Il1a*, (B) *Ilb*, (C), *Cxcl2*, (D) *Cd11b*, (E) *Nos2*, and (F) *Fcγr3* was calculated compared to PBS-treated control pups. Groups represent n = 7–11 animals with mean ±SEM. Significance is indicated by *, P < 0.05; **, P < 0.01; ***, P < 0.001.

Intranasal administration of recombinant Ply upregulated expression of *Il1b*, but not *Il1a*, (Fig 5A and 5B) and markers of phagocyte recruitment or activity, including *Cxcl2*, *Nos2*, *Fcγr3* (Fig 5C–5F). These increases in gene expression were not observed for toxoid PdB, deficient in pore-formation. These findings confirm the critical role of pneumolysin cytotoxicity in inducing IL-1 responses that promote clearance of otherwise persistent pneumococcal colonization.

### Capsule serotype affects persistence of *S*. *pneumoniae* colonization

**Fig 6:**
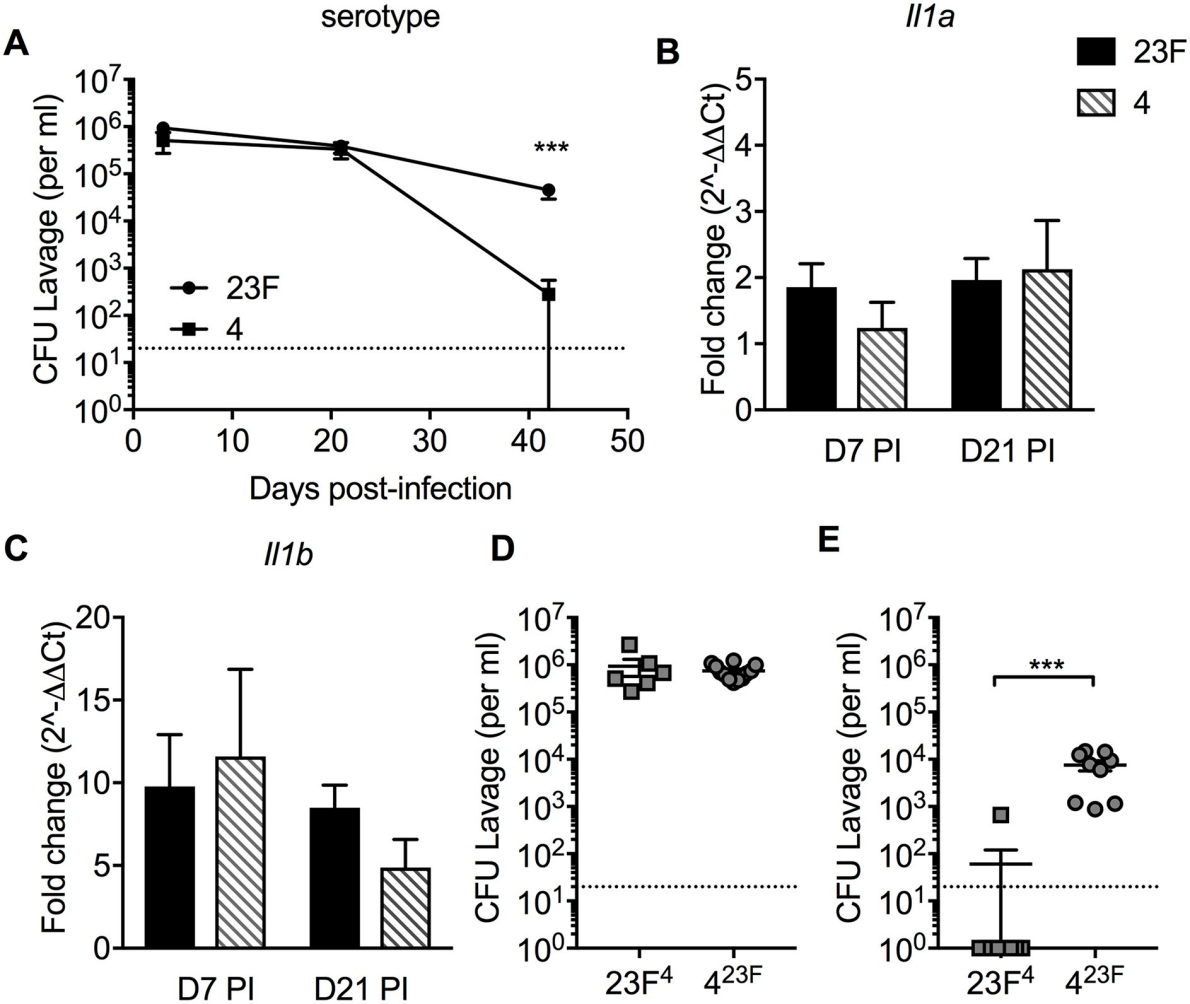
Capsule type determines serotype-dependent clearance. Pups at day 4 of life received i.n. infection with serotype 23F or 4 *S*. *pneumoniae*. (A) Pneumococcal colonization density at 3 and 6 weeks after infection. At day 7 (D7) and 21 (D21) after challenge, mucosal expression of (B) *Il1a* and (C) *Il1b* was measured by qRT-PCR as fold change compared to mock challenged age-controlled mice. (D-E) At day 4 of life, pups were i.n. infected with *S*. *pneumoniae* isogenic capsular switch mutants 23F^4^ (23F isolate expressing 4 CPS) and 4^23F^ (4 isolate expressing the 23F CPS). Pneumococcal colonization density was determined at (D) 5 days and (E) 6 weeks after inoculation. Groups represent n = 5–13 animals with mean ±SEM. Dotted line represents the lower limit of detection. Significance is indicated by ***, P < 0.001.

In Fig 1, we showed differences in the duration of colonization for two isolates differing in CPS (serotypes 4 and 23F) (Relevant data juxtaposed in Fig 6A). To determine whether this difference was due to IL-1 signaling, we compared the IL-1 stimulating capacity of the two isolates. We found that serotype 4 and 23F isolates induced similar expression of *Il1a*, *Il1b*, and IL-1 signaling related transcripts at 7 and 21 days post-challenge (Fig 6B and 6C, and S2 Fig), a result that could be due to similar levels of pneumolysin expression and hemolytic activity in these two isolates. To determine whether CPS type or bacterial genetic background is the determinant of isolate-specific persistence, we made use of capsule-switch strains of these two isolates [30, 45]. We infected pups at day 4 of life with the isogenic strains 23F^4^ (23F isolate expressing 4 CPS) and 4^23F^ (4 isolate expressing the 23F CPS). The capsule-switch strains colonized equivalently during early infection (Fig 6D). However, at 6 weeks post-challenge, the 4^23F^ strain persisted whereas the 23F^4^ strain was cleared (Fig 6E), demonstrating that strains carrying the 23F CPS are more persistent, regardless of genetic background. Thus, serotype-dependent differences in clearance appear to act through processes downstream or independent of IL-1 signaling.

### Age-related IL-1 responses during early pneumococcal colonization

**Fig 7:**
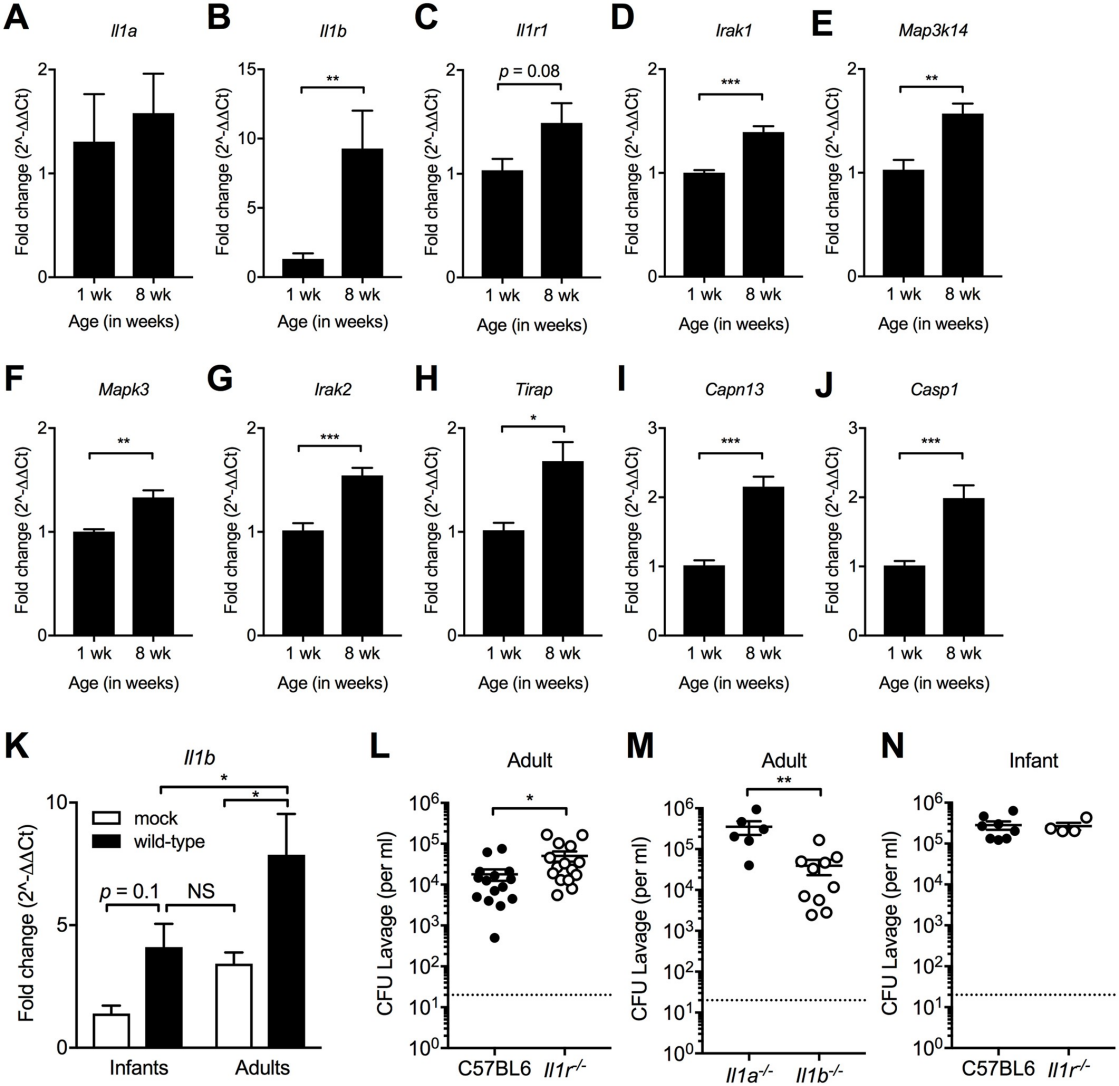
Repressed IL-1 signaling in infants. Five uninfected pups aged 1 week (Infant Mock) and five uninfected adults aged 8 weeks (Adult Mock) received RLT lavages from which RNA was subjected to qRT-PCR analysis. (A-J) Expression of (A) *Il1a*, (B) *Il1b*, (C) *Il1r1*, (D) *Irak1*, (E) *Map3k14*, (F) *Mapk3*, (G) *Irak2*, (H) *Tirap*, (I) *Capn13*, and (J) *Casp1* by qRT-PCR was calculated as fold change in uninfected adults (aged 8 weeks) as compared to uninfected infants (aged 1 week). (K) *Il1b* expression in uninfected (mock) and serotype 23F infected infants and adults calculated as fold-change compared to mock-infected infants. (L-N) Serotype 23F i.n. colonization of (L) C57BL6 and *Il1r*^*-/-*^ and (M) *Il1a*^*-/-*^ and *Il1b*^*-/-*^ adult mice (aged 8 weeks) and (N) C57BL6 and *Il1r*^*-/-*^ pups (aged 1 week). Pneumococcal colonization density in adult and infant URT was determined 3 days post-inoculation. Groups represent n = 4–15 animals with mean ±SEM. Significance is indicated by *, P < 0.05; **, P < 0.01.

Given the importance of IL-1 in clearance of otherwise persistent colonization, we questioned whether differences in IL-1 signaling underlie the age-dependent susceptibility to *S*. *pneumoniae* colonization. We previously showed that *Il1r*^*-/-*^ adult mice have reduced numbers of neutrophils during early colonization, fewer macrophages later in carriage, and prolonged bacterial colonization [19]. We used qRT-PCR to transcriptionally profile the IL-1 signaling pathway in the URT of uninfected infant (1 week of age) and adult (8 weeks of age) mice. As hypothesized, qRT-PCR confirmed significantly decreased expression of multiple IL-1-related signaling transcripts in uninfected infant compared to adult mice (Fig 7A–7J). Although colonization increased its expression, the level of *Il1b* expression in colonized infant mice only reached that of uncolonized adult mice (Fig 7K). In adult mice with increased IL-1 responses at baseline, colonization of *Il1r*^*-/-*^ mice at 3 days post-challenge resulted in impaired initial clearance, consistent with results of prior studies showing the importance of IL-1 signaling in adults at 14 days post-inoculation [19] (Fig 7L). Loss of IL-1α, but not IL-1β, led to impaired initial clearance in adult mice (Fig 7M). Thus, the importance of IL-1 signaling, and in particular, the role of IL-1α could account for why clearance in infancy is delayed until reaching adulthood. Accordingly, for infant mice with dampened IL-1 signaling at baseline there was no effect of the absence of the IL-1R at 3 days post-challenge (Fig 7N). As shown in Fig 3, stimulation of robust responses with recombinant IL-1 cytokines was sufficient to reduce colonization density in infants during early colonization, suggesting the defect in infants is not due to an inability to respond to IL-1 cytokines (Fig 3E and 3F). Together these results support that diminished IL-1 signaling during infancy enhances susceptibility to *S*. *pneumoniae* colonization.

## Discussion

Although epidemiological studies have shown that carriage of *S*. *pneumoniae* is significantly prolonged in young children, indicative of a commensal lifestyle, the mechanisms underlying pneumococcal persistence remain unknown. Here we showed that IL-1 signaling was required for efficient clearance but is relatively deficient in infants compared to adults. Prolonged colonization of *S*. *pneumoniae* was shown to be mediated by either loss of its sole toxin, pneumolysin, or through repression of the IL-1 pathway. While studies *in vitro* have associated pneumolysin expression with induction of IL-1 responses, our results provide *in vivo* evidence that pneumolysin drives IL-1 signaling through IL-1α to promote clearance of *S*. *pneumoniae* in the URT. Consequently, absence of pneumolysin or loss of the IL-1 signaling pathway, either by genetic ablation of the IL-1 receptor, IL-1α or young age, was sufficient to promote persistent pneumococcal colonization of the URT. Prompt clearance, therefore, does not appear to be initiated until IL-1 responses mature (>21 days of age) and is dependent on the presence of pneumolysin.

Several aspects of the effect of pneumolysin merit further comment. Pneumococcal colonization with the Ply point mutant deficient in pore-formation showed a clearance defect. However, a greater effect was observed for the Ply mutant unable to bind membrane cholesterol, an earlier step in its activity. Apparently, binding of Ply to membrane cholesterol also contributes to perturbation of cell homeostasis as proposed from *in vitro* studies and this impacts toxin-mediated clearance [46]. Without the toxin, there appears to be little stimulation of host responses that drive eventual clearance. Our findings are consistent with prior reports that the gradual process of Ply-mediated clearance requires a mild sustained phagocyte influx for clearance [19, 34]. This raises the question of why the organism expresses a toxin that precipitates its own clearance. Although the expression of Ply may be disadvantageous for *S*. *pneumoniae* colonization duration in the current host, inflammation induced by the toxin is necessary for pneumococcal shedding at levels sufficient for transmission to a new, susceptible host [18]. Some naturally circulating pneumococcal strains lack *ply* or express low or non-cytolytic Ply variants [47, 48]. These might have a deficit in transmission, but an advantage in carriage duration, although natural colonization dynamics of these strains have not yet been assessed. In Fig 3K we show that in absence of IL-1α the wild-type strain colonizes at lower levels than the *ply-* mutant, indeed suggesting immune mechanisms other than IL-1 also contribute to clearance.

A caveat of this study is that we were unable to measure colonization-induced differences in IL-1 protein in the URT, perhaps because of the mild, gradual nature of the inflammatory process requiring many weeks to months for clearance to complete. Our colonization model contrasts with more dramatic infections where such events transpire over days. Pulse dosing with recombinant Ply, however, was sufficient to facilitate more rapid clearance. *In vitro* studies have reported that during pneumococcal infection Ply can regulate IL-1 responses by inducing rapid cell death through necroptotic or pyroptotic pathways due to disruption of the cell membrane [49–51]. Recently, pore-forming toxins including Ply were found to trigger necroptosis, the major cell death pathway in respiratory epithelial cells, in mice and non-human primates during bacterial lung infection [50]. In *in vitro* macrophage models, Ply pore-formation also causes a passive release of alarmin IL-1α following rapid cell death [38, 52]. Our RNA-seq analyses show more functional clustering of cell death pathways, including necroptosis and apoptosis, following pneumococcal colonization. The IL-1α precursor protein, which is constitutively expressed in many cell types at the mucosal barrier, including both epithelial and myeloid lineages [38–40, 52, 53], does not require cleavage for binding to the IL-1 receptor [39, 54], thus Ply-mediated passive release of IL-1α precursor could directly activate IL-1 signaling. In contrast, IL-1β precursor expression is induced in response to TLR stimulation, TNFα, and IL-1 itself, and requires active processing in order to bind the IL-1 receptor [37]. Canonical IL-1β processing and release is mediated by caspase 1 following activation of an inflammasome [37, 55, 56]. In contrast, cell death induced by bacterial virulence factors that result in the release of IL-1α protein does not require the inflammasome but may depend on caspase-11 [57]. Despite the extensive evidence of Ply and necroptosis in activating the NLRP3 inflammasome, we observed that Ply-mediated clearance of pneumococcal colonization was NLRP3 independent [42–44, 51, 58]. This result, along with the lack of a contribution of TLR2 upstream of IL-1β is consistent with a dominant role for IL-1α, which does not require increased expression or the inflammasome for its activity [19]. Similar to IL-1β, signaling downstream of cytosolic sensing (via NOD2 or Type I Interferons) affects clearance in adult mice but showed no contribution in infant mice, which exhibit more persistent colonization [14, 36]. Ply-dependent cytosolic sensing may only occur following uptake by professional phagocytes and, as noted above, the recruitment of these is attenuated in infant compared to adult mice [17, 31]. By dosing with recombinant pneumolysin the lack of a secretion mechanism for the toxin would have been bypassed potentially allowing for direct effects on non-professional phagocytes, including epithelial cells.

Our study raises the question of how signaling downstream of IL-1α affects carriage. IL-1-dependent activation of chemokines from neighboring nonhematopoietic cells or tissue-resident macrophages triggers the recruitment and activation of inflammatory hematopoetic cells to the site of damage [39]. This in turns initiates a positive feedback loop whereby the recruited myeloid cells respond to the inflammatory process by the release of more IL-1 cytokines. This loop could explain why we observed increased transcription of IL-1β, a cytokine transcribed and released predominantly by cells of hematopoetic origin. We were unable to detect significant differences in numbers of neutrophils or monocytes/macrophages in URT lavages by flow cytometry, perhaps due to the relatively mild and prolonged nature of the inflammatory process. Additionally, IL-1 signaling downstream of IL-1α has potent effects on phagocytic cells. Transcriptional profiling during persistent pneumococcal colonization demonstrated pneumolyin-dependent upregulation of factors involved in both phagocyte recruitment and function. IL-1 signaling also contributes to the differentiation of IL-17^+^ T cells and could impact eventual Th17-mediated pneumococcal clearance when these responses mature [59].

Colonization persistence was also found to depend on serotype, which is in agreement with clinical observations of serotype-dependent colonization duration in young children [5]. Serotype-specific differences in colonization duration in our model were not attributable to differences in IL-1 signaling. Isolates of serotype 23F and 4, which are cleared in 9 and 6 weeks, respectively, that express equivalent amounts of Ply generated similar IL-1 responses. Differences in physical properties of the capsule type or its amount may affect processes downstream of IL-1 signaling, such as the deposition of opsonins (complement and antibody) or uptake by professional phagocytes as previously documented [4, 37, 60, 61].

The attenuation of IL-1 signaling during infancy could account for why initiation of clearance is delayed until mice are approximately 25 days of age. The association of young age with impaired IL-1 responses observed from our mouse model provides a possible mechanism for enhanced susceptibility to *S*. *pneumoniae* during early childhood. However, factors regulating IL-1 signaling during early life remain unknown. We have shown previously that infant mice have an elevated URT mucosal inflammatory profile that depends on the presence of a microbiota [31]. The high mucosal expression of chemokines in infants decreases the gradient for phagocyte recruitment to the airway lumen and delays clearance of colonizing pneumococci. The situation with IL-1 signaling is different as we measured reduced expression of genes in the IL-1 pathway at baseline as compared to adult mice. One possibility is that infants have blunted IL-1 responses to allow for acquisition and establishment of a stable URT microbiota. Alternatively, it seems plausible that the imbalanced infant microbiota could underlie the repressed URT expression of IL-1 signaling genes, or blunted IL-1α responses, and is responsible for increased susceptibility of infants to URT pathogens. Studies in adult mice have shown that the microbiota affects expression of IL-1β precursor protein and is important for regulation of mucosal defense; whereas the impact of the microbiota on IL-1α-dependent effects have yet to be explored [62, 63]. Alternatively, metabolic and nutritional differences with age could affect mucosal inflammatory responses directly or indirectly through temporal changes in the microbiota [64].

The importance of IL-1 signaling in clearance of otherwise persistent colonization may have broad relevance to other systems [65–68]. IL-1α and IL-1β were found to have overlapping and non-redundant roles in bacterial clearance during lung infection with *Legionella pneumophila* [57]. Lack of IL-1 signaling, specifically IL-1α, during mycobacterial lung infection led to an inability to control bacterial replication and earlier mortality [66]. Additionally, both IL-1α and IL-1β were necessary for phagocyte recruitment and function to control lung infection of *Aspergillus fumigatus* [68]. The importance of intact and robust IL-1 responses is further reflected by the increase in severe bacterial infections seen in individuals with inborn errors involving IL-1 signaling or in patients receiving anti-IL-1 treatment for inflammatory diseases [69–73]. In particular, deficiencies in IL-1 signaling, including in IRAK4, Myd88 and NEMO, are associated with susceptibility to severe and recurrent pneumococcal infections during childhood [69, 70]. The elderly also suffer from frequent pneumococcal disease. Pneumococcal colonization is prolonged in aged mice [72–75], and *in vitro* and *in vivo* studies demonstrate reduced IL-1 protein release by aged human and mouse monocytes, and in the aged lung, following infection with *S*. *pneumoniae* [76, 77]. Differences in IL-1 signaling, therefore, may be relevant to a number of vulnerable populations.

## Supporting information

S1 Fig Group variance in infant URT samples used for RNA-seq. Principle Component Analysis (PCA) illustrating the variance between URT samples within the groups tested: mock- (Infant_Mock_D21), serotype 23F wild-type (Infant_WT_D21), and *ply-* (Infant_PLY_D21) infected infants 21 days post-colonization.
(TIF)

S2 Fig Serotype-induced expression of IL-1 related genes in the infant URT. Pups at day 4 of life received i.n. infection with serotype 23F or 4 *S*. *pneumoniae*. At day 7 (D7) after colonization, mucosal expression of (A) *Il1rn*, (B) *Irak2* and (C) *Tirap* was measured by qRT-PCR as fold change compared to mock infected age-controlled mice. Groups represent n = 11 animals.
(TIFF)

S1 Table Primer sequences for host genes used in this study. (DOCX)
