# Supplementary figures and images for: Age-related differences in IL-1 signaling and capsule serotype affect persistence of Streptococcus pneumoniae colonization
Source: PLoS Pathog. 2018 Oct 31;14(10):e1007396. doi: 10.1371/journal.ppat.1007396 (PMC6231672; doi:10.1371/journal.ppat.1007396)

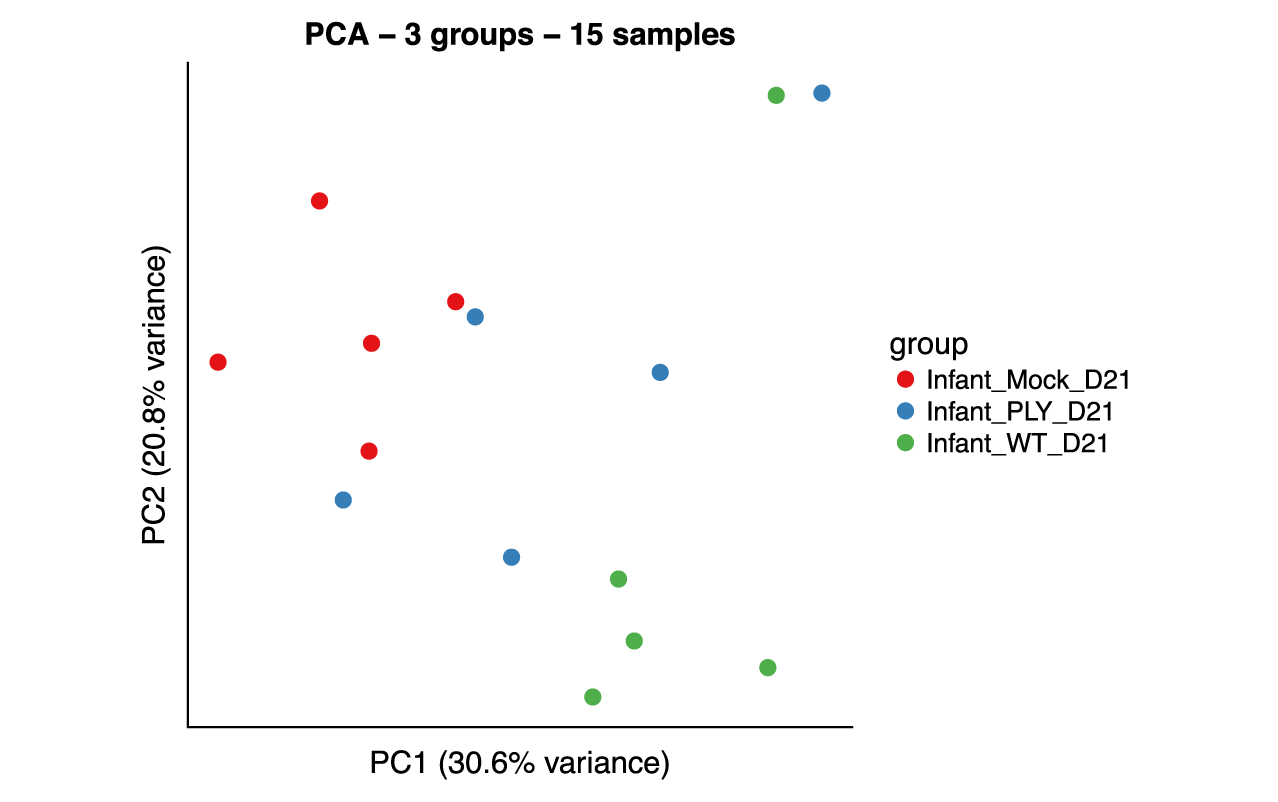

Supplement: S1 Fig — Principle Component Analysis (PCA) illustrating the variance between URT samples within the groups tested: mock- (Infant_Mock_D21), serotype 23F wild-type (Infant_WT_D21), and ply- (Infant_PLY_D21) infected infants 21 days post-colonization. (TIF) [file ppat.1007396.s001.tif]

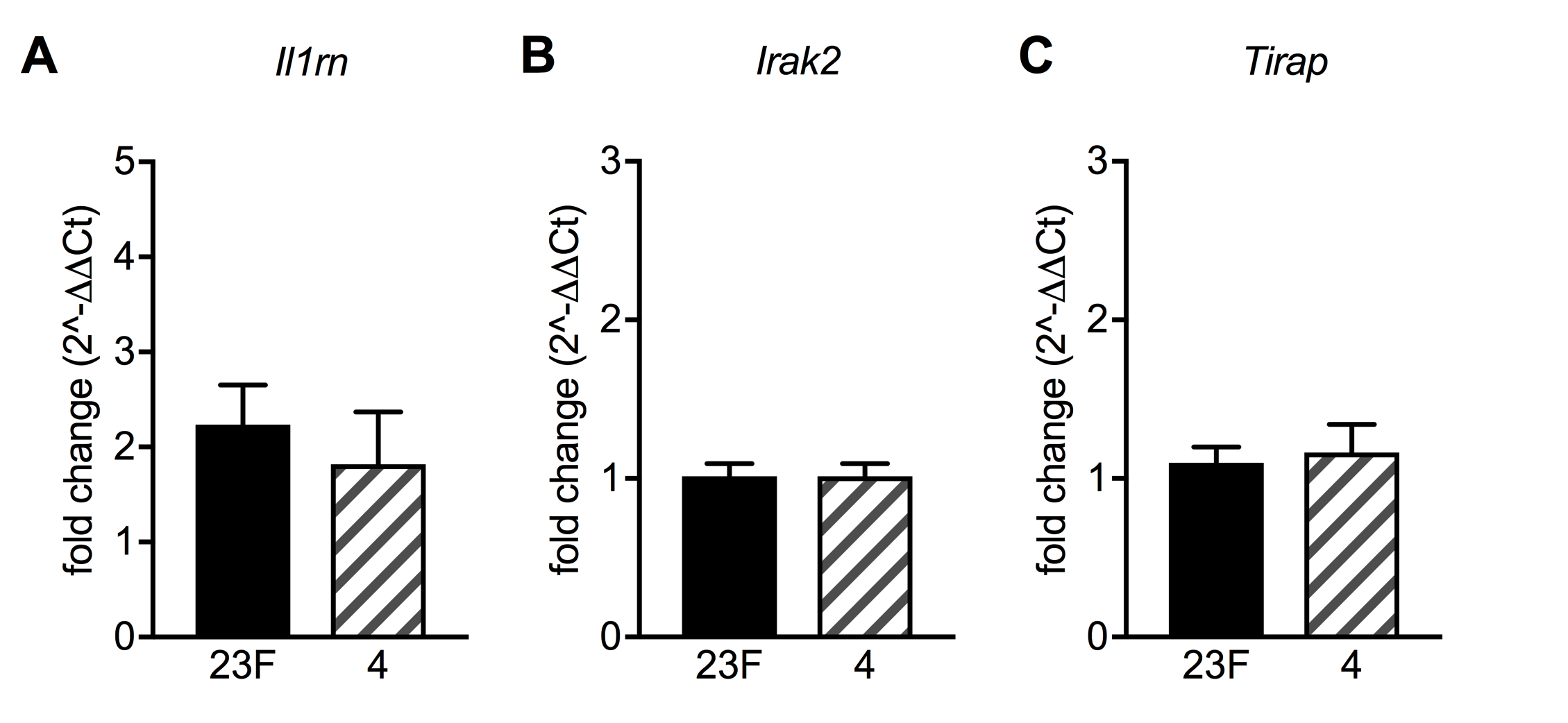

Supplement: S2 Fig — Pups at day 4 of life received i.n. infection with serotype 23F or 4 S. pneumoniae. At day 7 (D7) after colonization, mucosal expression of (A) Il1rn, (B) Irak2 and (C) Tirap was measured by qRT-PCR as fold change compared to mock infected age-controlled mice. Groups represent n = 11 animals. (TIFF) [file ppat.1007396.s002.tiff]
