# Supplementary material for: Age-related differences in IL-1 signaling and capsule serotype affect persistence of Streptococcus pneumoniae colonization
Source: PLoS Pathog. 2018 Oct 31;14(10):e1007396. doi: 10.1371/journal.ppat.1007396 (PMC6231672; doi:10.1371/journal.ppat.1007396)
Supplement: S1 Table — (DOCX) [file ppat.1007396.s003.docx]

**Age-related Differences in IL-1 Signaling and Capsule Serotype Affect Persistence of *Streptococcus pneumoniae* Colonization**

Kirsten Kuipers^1^, Kristen L. Lokken^1^, Tonia Zangari^1^, Mark A. Boyer^2^, Sunny Shin^2^, Jeffrey N. Weiser^1^

**Supporting Information**

**S1 Table Primer sequences for host genes used in this study**

| Gene | Primer sequence (5’ – 3’) | Gene | Primer sequence (5’ – 3’) |
| --- | --- | --- | --- |
| *gapdh* | Forward: AGGTCGGTGTGAACGGATTTG | *FcγR3* | Forward: CAGAATGCACACTCTGGAAGC |
|  | Reverse: TGTAGACCATGTAGTTGAGGTCA |  | Reverse: GGGTCCCTTCGCACATCAG |
| *Il17a* | Forward: GCTCCAGAAGGCCCTCAGA | *Il1r1* | Forward: GTGCTACTGGGGCTCATTTGT |
|  | Reverse: AGCTTTCCCTCCGCATTGA |  | Reverse: GGAGTAAGAGGACACTTGCGAAT |
| *Il1a* | Forward: ATGACCTGC AACAGGAAGTAAAA | *Irak1* | Forward: CAGAACCACCACAGATCATCATC |
|  | Reverse: TGTGATGAGTTTTGGTGTTTCTG |  | Reverse: AGGCTTCAATTCCAATAGCATCA |
| *Il1b* | Forward: GCAACTGTTCCTGAACTCAACT | *Map3k14* | Forward: TGTGGGAAGTGGGAGATCCTA |
|  | Reverse: ATCTTTTGGGGTCCGTCAACT |  | Reverse: GGCTGAACTCTTGGCTATTCTCA |
| *Ccl22* | Forward: AGGTCCCTATGGTGCCAATGT | *Mapk3* | Forward: TCCGCCATGAGAATGTTATAGGC |
|  | Reverse: CGGCAGGATTTTGAGGTCCA |  | Reverse: GGTGGTGTTGATAAGCAGATTGG |
| *Il1rn* | Forward: GCTCATTGCTGGGTACTTACAA | *Irak2* | Forward: GGAAGCCGGTTCCTGAGAG |
|  | Reverse: CCAGACTTGGCACAAGACAGG |  | Reverse: GGCCGGACTTTCTCCTGTTC |
| *Cxcl2* | Forward: CCACCAACCACCAGGCTAC | *Tirap* | Forward: CCTCCTCCACTCCGTCCAA |
|  | Reverse: GCTTCAGGGTCAAGGGCAAA |  | Reverse: CTTTCCTGGGAGATCGGCAT |
| *Cd11b* | Forward: ATGGACGCTGATGGCAATACC | *Capn13* | Forward: TCTGGAAGGTGCAAGCAGATT |
|  | Reverse: TCCCCATTCACGTCTCCCA |  | Reverse: TGGTGCGAATAGCTTTGGTCC |
| *Nos2* | Forward: TTGGGTCTTGTTCACTCCACGG | *Casp1* | Forward: ACAAGGCACGGGACCTATG |
|  | Reverse: CCTCTTTCAGGTCACTTTGGTAGG |  | Reverse: TCCCAGTCAGTCCTGGAAATG |
